# Supplementary material for: People are surprisingly hesitant to reach out to old friends
Source: Commun Psychol. 2024 Apr 23;2:34. doi: 10.1038/s44271-024-00075-8 (PMC11332216; doi:10.1038/s44271-024-00075-8)
Supplement: Supplementary file 1 — Supplementary Information [file 44271_2024_75_MOESM1_ESM.pdf]

Supplementary Information

Supplementary Notes

This document contains two key sections. First, it contains additional measures and exploratory analyses that were not reported in the main text for the seven main studies. Second, it contains the methods and results of two additional studies (Supplementary Studies S8 and S9). As with the studies reported in the main text, the materials, data and code for these additional studies are available here: https://osf.io/kydb3, along with a file that has links to the pre-registrations.

Supplementary Methods

Study 2

In addition to the measures presented in the main text, we also asked participants to what extent they would be happy to reach out to [hear from] their old friend for each of the same reasons listed in Study 1 (e.g., on their/your birthday). Responses were provided on a scale ranging from 1 = *not at all to* 7 = *extremely*.

We asked participants to rate the extent to which seven barriers would affect their willingness to reach out to [hear from] their old friend (1 = *not at all relevant* to 7 = *extremely relevant*). In the *reaching out* condition, we used the same barriers as in Study 1. In the *hearing from* condition, the last two barriers (don't know if the other person is interested; don't want to bother them) were omitted because they did not make sense.

Results. Consistent with our pre-registered prediction, independent samples t-tests indicated that participants saw all reasons as more justifiable when hearing from (vs. reaching out to) an old friend (see Supplementary Figure 1), birthday: $M_{hear\text{-}from}$ = 5.3, SD = 1.6; $M_{reach\text{-}out}$ = 4.8, SD = 1.8; $t(196)$ = 1.88, one-tailed p = .03, d = .27, ΔM = 0.46, CI_{95} = [-0.02, 0.93];

reminder of shared experience: $M_{hear\text{-}from}$ = 5.7, SD = 1.6; $M_{reach\text{-}out}$ = 4.4, SD = 1.9; $t(197) = 5.37$, one-tailed $p < .001$, $d = .76$, $\Delta M = 1.32$, CI_{95} = [0.83, 1.80]; on a holiday: $M_{hear\text{-}from}$ = 5.1, SD = 1.7; $M_{reach\text{-}out}$ = 4.2, SD = 1.9; $t(197) = 3.84$, one-tailed $p < .001$, $d = .54$, $\Delta M = 0.98$, CI_{95} = [0.48, 1.49]; thinking about them: $M_{hear\text{-}from}$ = 5.6, SD = 1.6; $M_{reach\text{-}out}$ = 3.9, SD = 1.8; $t(197)$ = 7.05, one-tailed $p < .001$, $d = 1.00$, $\Delta M = 1.70$, CI_{95} = [1.22, 2.17]; in their neighbourhood: $M_{hear\text{-}from}$ = 4.9, SD = 1.7; $M_{reach\text{-}out}$ = 3.8, SD = 2.0; $t(197) = 3.97$, one-tailed $p < .001$, $d = .56$, ΔM = 1.06, CI_{95} = [0.53, 1.59]; just because: $M_{hear\text{-}from}$ = 5.6, SD = 1.6; $M_{reach\text{-}out}$ = 3.9, SD = 1.8; $t(197)$ = 7.08, one-tailed $p < .001$, $d = 1.00$, $\Delta M = 1.72$, CI_{95} = [1.24, 2.19]; saw something they might enjoy: $M_{hear\text{-}from}$ = 5.4, SD = 1.7; $M_{reach\text{-}out}$ = 3.7, SD = 2.0; $t(197) = 6.74$, one-tailed $p < .001$, d = .96, $\Delta M = 1.73$, CI_{95} = [1.23, 2.24]; to ask for a favour: $M_{hear\text{-}from}$ = 3.8, SD = 1.9; $M_{reach\text{-}out}$ = 2.8, SD = 1.7; $t(197) = 4.06$, one-tailed $p < .001$, $d = .58$, $\Delta M = 1.03$, CI_{95} = [0.53, 1.53]. Notably, the (descriptively) smallest difference observed across conditions was regarding birthdays because, as observed in Study 1, participants reported the greatest willingness to reach out in this context.

Supplementary Figure 1. *Endorsement of various reasons for reaching out to or hearing from old friends in Study 2*

Boxplot showing all the data; barring missing data, all participants (N = 199) rated each item. The upper and lower hinges of the boxplot correspond to the first and third quartiles (the 25th and 75th percentiles). The median is indicated by the line in the boxplot, and the mean is indicated by the blue diamond.

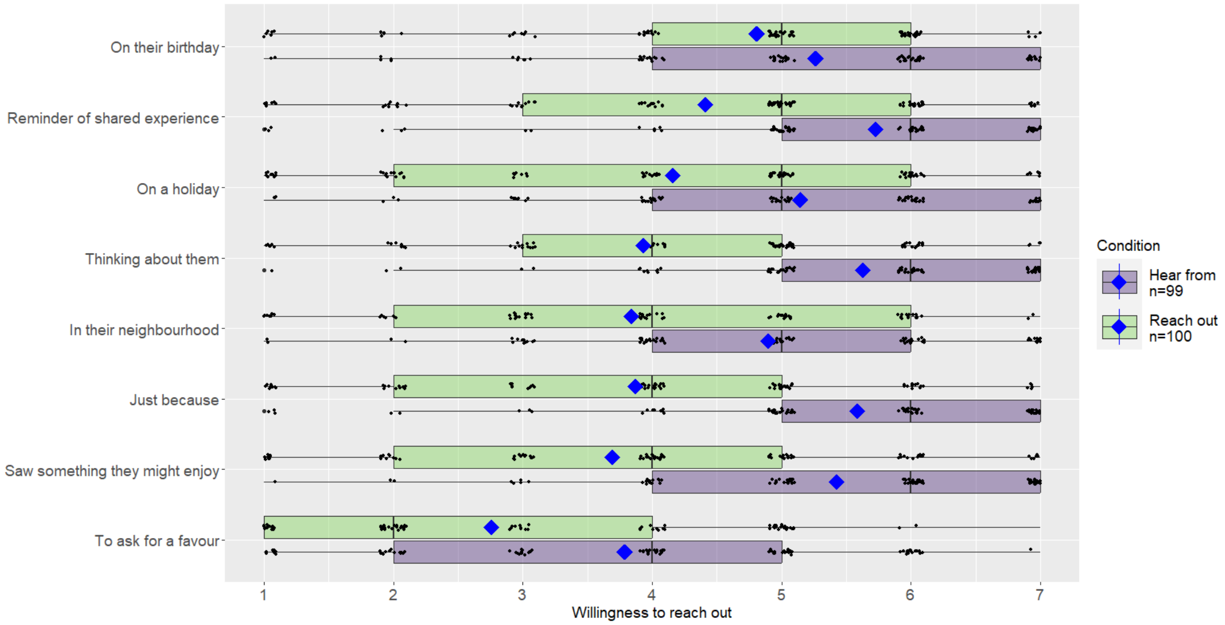

We ran exploratory independent samples t-tests to examine whether participants rated barriers as less relevant in the hearing from (vs. reaching out) condition. There was little evidence for differences between conditions (see Supplementary Figure 2). Two barriers were rated as less relevant in hearing from (vs. reaching out) condition: being awkward to reconnect after all this time ($M_{hear\text{-}from}$ = 4.5, *SD* = 1.9; $M_{reach\text{-}out}$ = 5.4, *SD* = 1.8; *t*(197) = -3.27, one-tailed *p* < .001, *d* = -.46, ΔM = -0.86, CI_{95} = [-1.37, -0.34]) and worrying about getting the wording of the message just right ($M_{hear\text{-}from}$ = 3.6, *SD* = 2.0; $M_{reach\text{-}out}$ = 4.4, *SD* = 2.0; *t*(197) = -2.97, one-tailed *p* = .002, *d* = -.42, ΔM = -0.82, CI_{95} = [-1.37, -0.28]). The differences between the remaining barriers were not significant: not having anything important to say ($M_{hear\text{-}from}$ = 3.6, *SD* = 2.0; $M_{reach\text{-}out}$ = 4.0, *SD* = 2.0; *t*(195) = -1.49, one-tailed *p* = .07, *d* = -.21, ΔM = -0.43, CI_{95} = [-1.00, 0.14]); not having time for a longer catch-up ($M_{hear\text{-}from}$ = 3.7, *SD* = 1.9; $M_{reach\text{-}out}$ = 3.9, *SD* = 2.0; *t*(196) = -1.00, one-tailed *p* = .16, *d* = -.14, ΔM = -0.28, CI_{95} = [-0.82, 0.27]); being too busy

($M_{hear\text{-}from}$ = 3.5, SD = 1.9; $M_{reach\text{-}out}$ = 3.9, SD = 1.9; t(197) = -1.46, one-tailed p = .07, d = -.21, ΔM = -0.40, CI_{95} = [-0.93, 0.14])

Supplementary Figure 2. *Endorsement of various barriers to reaching out to and hearing from old friends in Study 2*

Boxplot showing all the data; barring missing data, all participants (N = 199) rated each item. The upper and lower hinges of the boxplot correspond to the first and third quartiles (the 25th and 75th percentiles). The median is indicated by the line in the boxplot, and the mean is indicated by the blue diamond.

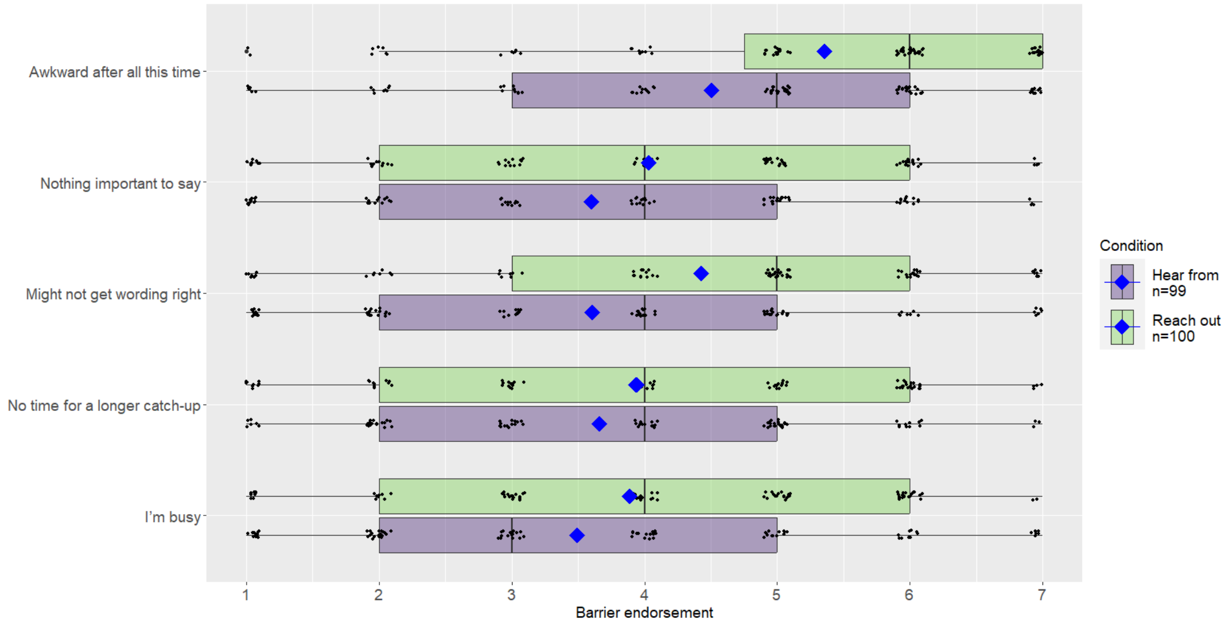

Study 3

We asked participants to rate the extent to which various personal and prosocial motivations were relevant to their decision to reach out: (i) it's a nice thing to do, (ii) it would make them happy, (iii) it would make their old friend happy, (iv) they miss their old friend, (v) expected regret if they didn't reach out (α = .84). Responses were provided on a scale ranging from 1 = *not at all relevant* to 7 = *extremely relevant*.

In addition to the seven barriers included in Studies 1 and 2, we included new barriers: (viii) guilt at having lost touch, (ix) worried that their old friend would think they had an ulterior motive, (x) worried that their old friend would think they were lonely, (α = .79). Participants rated how relevant each barrier was, using a scale ranging from 1 = *not at all relevant* to 7 = *extremely relevant*.

Results. The means for each of the personal and prosocial motivations were (descriptively) above the midpoint of the scale (see Supplementary Figure 3).

Supplementary Figure 3. *Endorsement of various motivations for reaching out to an old friend in Study 3*

Boxplot showing all the data; barring missing data, all participants (*N* = 453) rated each item. The upper and lower hinges of the boxplot correspond to the first and third quartiles (the 25th and 75th percentiles). The median is indicated by the line in the boxplot, and the mean is indicated by the blue diamond.

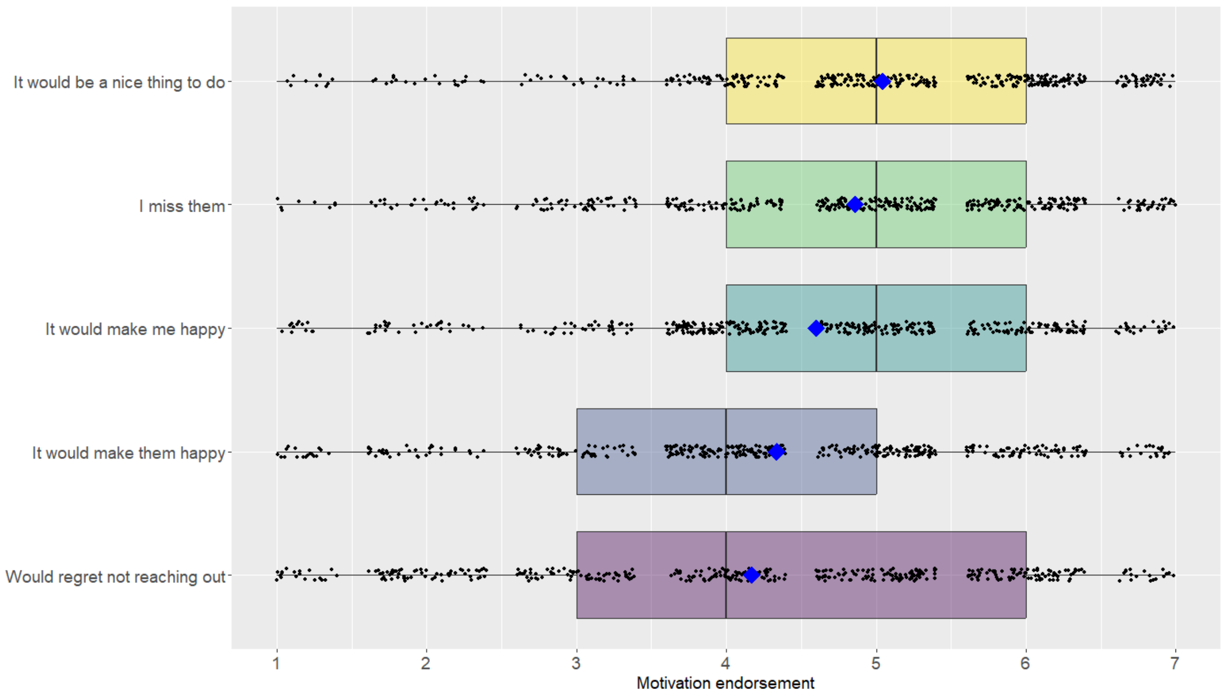

In terms of barriers, as in Studies 1 and 2, participants were most likely to agree with statements that suggested the recipient may be uninterested, or that it would be awkward to reach out (see Supplementary Figure 4). They endorsed the idea of feeling guilty about having lost touch (descriptively) more than any of the barriers that were included in Studies 1 and 2. Participants were less likely to endorse reasons about practical constraints, such as limits on their time.

Participants who chose to reach out to their old friend (vs. those who chose not to reach out) reported considering the barriers less relevant ($M = 3.3$, $SD = 1.1$ vs. $M = 3.9$, $SD = 1.1$), $t(451) = 4.70$, two-tailed $p < .001$, $d = .49$, $\Delta M = 0.55$, $CI_{95} = [0.32, 0.78]$, and the motivations more relevant ($M = 5.4$, $SD = 0.9$ vs. $M = 4.3$, $SD = 1.3$), two-tailed $t(451) = -9.04$, $p < .001$, $d = -.95$, $\Delta M = -1.10$, $CI_{95} = [-1.34, -0.86]$.

Supplementary Figure 4. *Endorsement of various barriers to reaching out to an old friend in Study 3*

Boxplot showing all the data; barring missing data, all participants ($N = 453$) rated each item. The upper and lower hinges of the boxplot correspond to the first and third quartiles (the 25th and 75th percentiles). The median is indicated by the line in the boxplot, and the mean is indicated by the blue diamond.

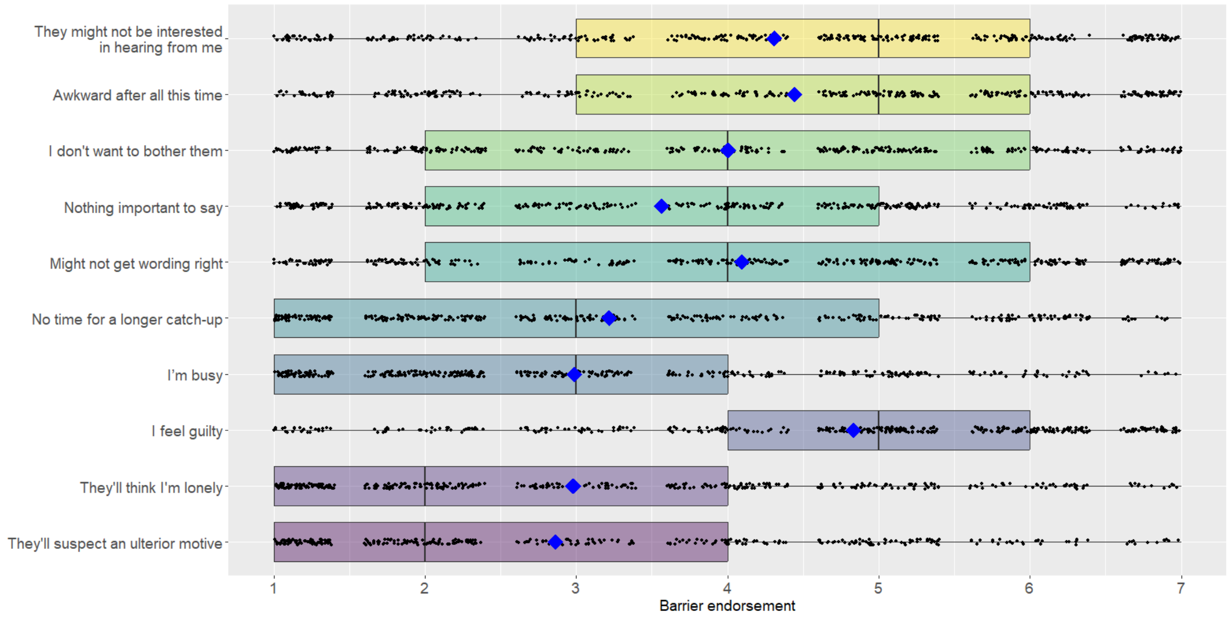

Study 4

We asked participants how much they had considered several barriers while making their decision: (i) having nothing important to say, (ii) not having time for a longer catch-up, (iii) worrying that the target would not be interested in hearing from them, (iv) worrying that the target might not respond. We included only these four barriers because we reasoned that they were likely to be affected by the manipulations. Responses were provided on a 7-point Likert scale ranging from *1 = not at all relevant* to *7 = extremely relevant*.

Results. We tested whether there were differences between conditions in how likely people were to endorse the various barriers. There were no differences between conditions in not having time for a longer catch-up, $F(2, 601) = 1.72$, $p = .18$, worrying that their estranged friend would not be interested in hearing from them, $F(2, 601) = 0.28$, $p = .75$, or worrying that their estranged friend would not respond, $F(2, 601) = 0.62$, $p = .54$. There were, however, differences in worrying about having nothing important to say, $F(2, 601) = 6.43$, $p = .002$. Follow-up paired comparisons, using a Tukey's test, revealed that people in the control condition ($M = 3.9$, $SD =$

2.1) were more worried than people in the message condition (*M* = 3.3, *SD* = 2.0), $p = .008$, and the message plus encouragement condition (*M* = 3.3, *SD* = 1.7), $p = .004$.

Study 5

Results. As in Study 1, a significant majority (78.4%) of participants reported that they had lost touch with a friend they care about, $X^2(1) = 92.58$, $p < .001$. Of these individuals, a significant majority (87.9%) said that they had thought about reaching out to a friend they had lost touch with but did not do so, $X^2(1) = 129.02$, $p < .001$.

Exploratory paired-samples t-test analyses indicated that, unsurprisingly, people were *less* willing to reach out to an old friend than they were to listen to a beloved song or eat ice cream, $t(287) = -14.55$, two-tailed $p < .001$, $d = .86$, and $t(287)= -6.03$, two-tailed $p < .001$, $d = .36$, respectively. Also unsurprisingly, people were *more* willing to reach out to an old friend than to book a physical or dental appointment or sort a bag of coins, $t(287) = 2.72$, two-tailed $p = .01$, $d = .16$, and $t(287) = 4.34$, two-tailed $p < .001$, $d = .26$, respectively. People were no more willing to reach out to an old friend than they were to hold a hand grip for 30 sec or pick up litter, $t(287) = -1.93$, two-tailed $p = .06$, $d = .11$, and $t(287) = -1.09$, two-tailed $p = .28$, $d = .06$, respectively.

Study 6

Results. Participants indicated the type of relationship they had with each target, and how they knew each target (i.e., the source of their relationship). For full descriptives on the type and source of relationships for all targets (i.e., old friends, current friends, new acquaintances), see Supplementary Figures 5 and 6.

Supplementary Figure 5. *Proportion of targets (old friends, current friends, new acquaintances) who reflect each relationship type in Study 6*

Bar graph showing the proportion of nominated targets (*n* = 1391 old friends, *n* = 349 current friends, *n* = 322 new acquaintances) that matched each relationship type.

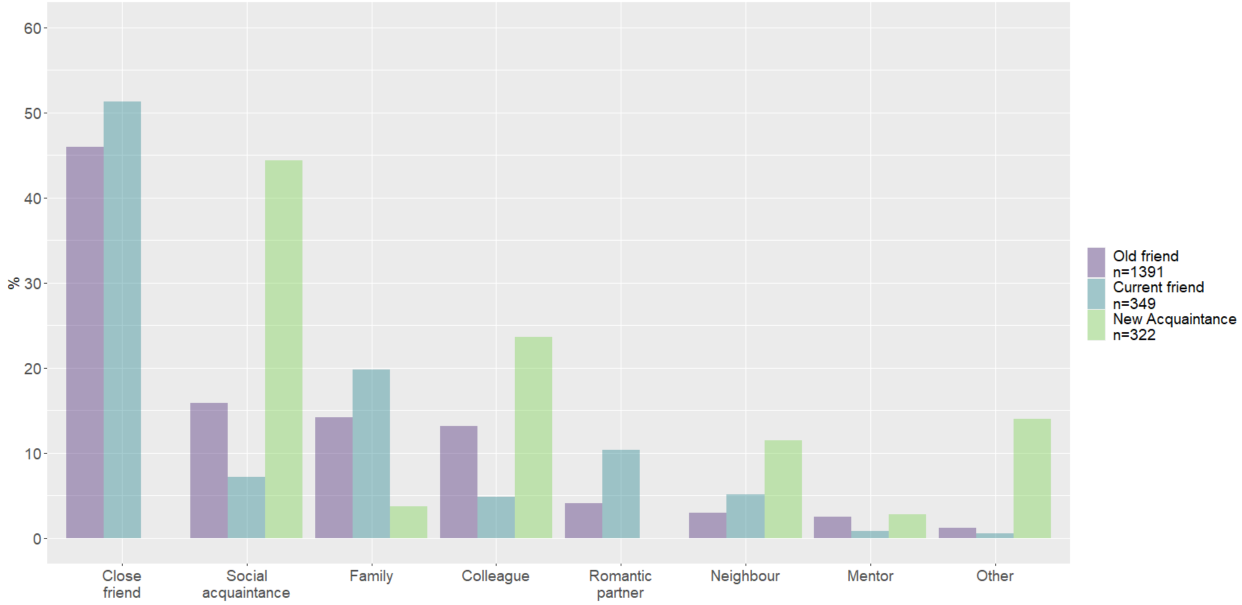

Supplementary Figure 6. *Proportion of targets (old friends, current friends, new acquaintances) who reflect each relationship source in Study 6*

Bar graph showing the proportion of nominated targets (*n* = 1456 old friends, *n* = 361 current friends, *n* = 330 new acquaintances) that matched each relationship source.

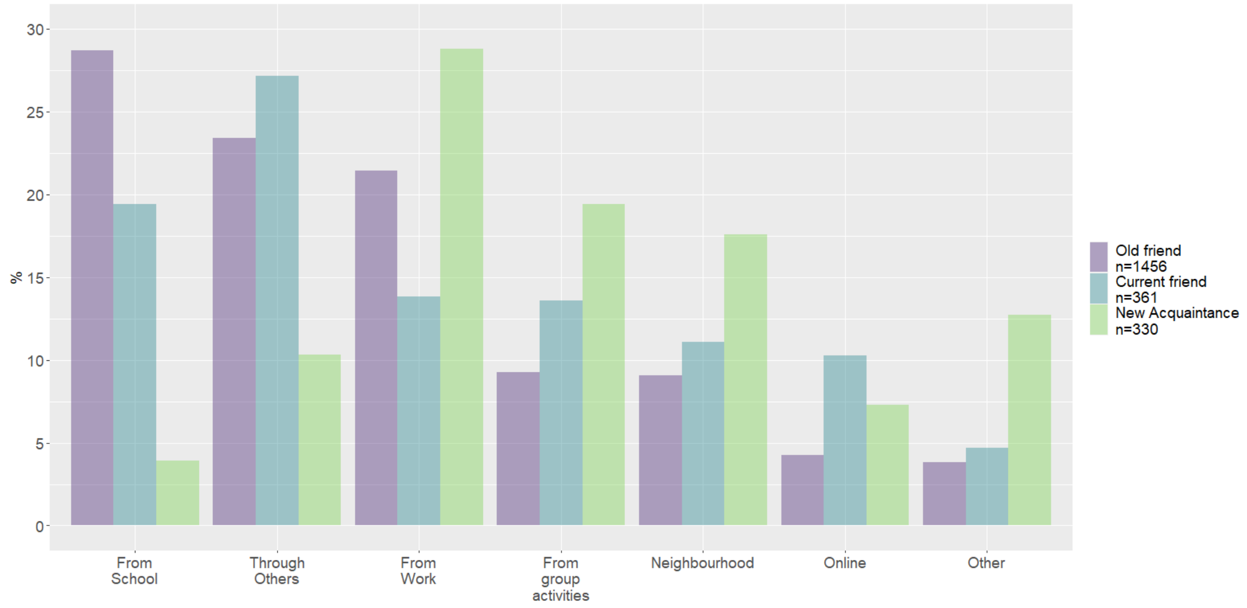

Although our main analyses focused on old friends, we also asked participants to nominate additional targets to allow us to benchmark feelings of familiarity and recency of contact. Specifically, we asked participants to identify and rate both their familiarity and recency of contact with one current friend (which we assumed would be high familiarity, high recency) and one new acquaintance (which we assumed to be low familiarity, high recency). Consistent with our intuitions, an exploratory one-way ANOVA with follow-up paired comparisons (Bonferroni-corrected to adjust for multiple comparisons) revealed that people had been in touch with their old friends (M = 1.8, SD = 0.7) less recently than their current friend (M = 4.4, SD = 1.0) or their new acquaintance (M = 3.9, SD = 1.0), $F(2, 634) = 754.36$, $p < .001$, $\eta_p^2 = .70$. Additionally, current friends (M = 5.6, SD = 1.1) were more familiar than old friends (M = 3.7, SD = 1.2), who were more familiar than new acquaintances (M = 2.5, SD = 1.4), $F(2, 634) =$ 645.93, $p < .001$, $\eta_p^2 = .67$. There was also a main effect of target on willingness to reach out, $F(2, 634) = 305.00$, $p < .001$, $\eta_p^2 = .49$. Participants were more willing to reach out to current

friends ($M = 6.3$, $SD = 1.0$) than to old friends ($M = 4.6$, $SD = 1.5$), $p < .001$, and more willing to reach out to old friends than to new acquaintances ($M = 3.8$, $SD = 1.9$), $p < .001$.

An exploratory linear mixed model revealed that recency also predicted willingness to reach out, $b = .66$, $SD = .04$, 95% CI = [0.58, 0.75], $t = 15.11$. Similarly, when we averaged recency across all old friends, participants who had been in touch with their old friends more recently were more willing to reach out to their old friends, $r(319) = .39$, $p < .001$. Recency was also correlated with willingness to reach out to current friends, $r(319) = .23$, $p < .001$, but not new acquaintances, $r(318) = .11$, $p = .06$.

Study 7

Results. People who reached out reported the barriers as being less relevant ($M = 3.5$, $SD = 1.2$) and the motivators as being more relevant ($M = 4.6$, $SD = 1.4$) than did people who did not reach out ($M = 4.1$, $SD = 1.1$; $M = 4.0$, $SD = 1.2$), $t(186) = 3.32$, one-tailed $p < .001$, $d = .49$, $\Delta M = 0.54$, CI_{95} = [0.22, 0.87] and $t(185) = 3.43$, one-tailed $p < .001$, $d = -.51$, $\Delta M = -0.63$, CI_{95} = [-0.99, -0.27], respectively.

Supplementary Study S8

The seven studies reported in the main text demonstrate that people are generally reluctant to reach out to old friends. One reason for this may be because people assume that others are willing to reach out instead, thereby relieving them of the task. In Supplementary Study 8 we explored this possibility by examining whether people overestimate the proportion of others who are willing to reach out to an old friend.

Method

Participants. Six-hundred thirty-seven American adults ($M_{age} = 43.8$, $SD = 17.3$; 331 women, 304 men, 2 other) recruited by Dynata completed this survey as part of a separate study.

The sample was representative of the United States population in terms of age, ethnicity, gender, region, and household income. Sample size was determined by a power calculation for the separate investigation.

Procedure

Participants were asked to read a simplified description of the control condition in Study 3:

> *Imagine that 10 people were asked to name a friend they had lost touch with. This friend was someone they wanted to reconnect with and thought would be happy to hear from them. Imagine these 10 people typed a message to their old friend, and were encouraged to send it.*

Because some people may be unfamiliar or uncomfortable with percentages, we simplified the scenario by asking participants to estimate how many people out of a possible 10 would reach out to an old friend. Specifically, we asked "How many of the 10 people do you think would actually send the message?" Responses were provided on a 0-10 slider scale. Afterward, we multiplied the response by 10 to get a percentage, which we compared to the actual frequency.

We predicted that participants would estimate that the rate of reaching out would be higher than the actual rate observed in Study 3. We note that the pre-registration describes two predictions, but ultimately, we were only able to examine one due to a survey programming error.

Results

On average, participants predicted that 5.66 people out of 10 (56.6%) would reach out to an old friend. We compared this estimate to 30% (we rounded up from the actual percentage

observed in Study 3: 27.5%) with a one-sample t-test. Consistent with our pre-registered prediction, participants significantly overestimated the likelihood of people reaching out to an old friend, $t(636) = 29.03$, one-tailed $p < .001$, $d = 1.15$. The estimate of 5.66 (or 56.6%) was also significantly higher than the reaching out rate observed in the control condition in Study 4 (42.5%), $t(636) = 15.39$, one-tailed $p < .001$. Thus, participants expected a greater proportion of people to reach out to an old friend than those who actually did. This finding is consistent with the possibility that people hope or expect others to reach out when relationships fade. But are people also overly optimistic about their own ability to reach out?

Supplementary Study S9

Study S8 finds that people are inaccurate about how likely *others* are to reach out to an old friend, such that most people tend to predict that a greater percentage of people will reach out than actually do. But are people also overly optimistic about their own reaching out behaviours?

Method. A total of 628 participants were recruited in public spaces on a university campus in Canada in exchange for candy. As required by the local ethics board at the site of data collection, participants were asked before the study to provide informed consent for participation and, separately, to grant permission to share their responses in an online repository for open science initiatives. We report results from the sample of 598 participants who gave permission to share their data, so that these findings can be replicated with the file posted on the OSF. Findings do not differ in the full sample. Of the 598 participants, 371 individuals said we could re-contact them with a reminder and follow-up study one week later. Ultimately, 191 of these individuals ($M_{age} = 21.2$, $SD = 5.0$; 120 women, 61 men, 9 other, and 1 participant with undisclosed gender) replied to the follow-up survey and therefore constitute our final sample. This final sample

surpasses our pre-registered target sample of 182 participants needed to provide 85% power to detect a small size effect (*dz* = .2) with a paired samples t-test and one-tailed alpha at .05.

Participants completed a short online survey in which they were given a brief description of old friends in which they were told that "life can be busy" and "sometimes we lose touch with people we like." Afterward, participants were told that recent research suggests that reaching out to old friends can have benefits for both parties, such as increased happiness for the actor and feelings of appreciation for the recipient. In light of this evidence, we asked participants to consider reaching out to old friends with whom they had lost touch. Specifically, participants were asked to indicate how many old friends they could commit to reaching out to in the coming week via phone, text, email, social media, or in person on a 0-100 scale. Participants were then asked if we could re-contact them with a reminder and brief follow-up survey in one week (yes/no) and, if yes, what was the best email to reach them on. Finally, participants reported their demographic information.

Three days after completing the baseline survey, participants who agreed to the follow-up were sent a reminder email saying "Have you remembered to reach out to an old friend this week? Research suggests that reaching out to people we like but with whom we have lost touch can have benefits – for you and them!"

Four days after the reminder email (i.e. one week after completing the baseline survey), participants who agreed to the follow-up were sent a brief survey asking how many old friends they had reached out to over the past week. Responses were provided on the same 0-100 scale and, if participants reported a number larger than zero, they were asked to briefly describe how reaching out went, in an open-ended textbox.

We pre-registered one confirmatory hypothesis: we predicted that, on average, people would overestimate how many old friends they would reach out to in the coming week.

Results. A paired samples t-test revealed that participants reported reaching out to significantly fewer friends than they intended, $t(190) = 5.32$, one-tailed $p < .001$. Specifically, participants estimated reaching out to an average of more than eight old friends in the coming week during the baseline survey ($M = 8.6$, $SD = 16.3$) but when they completed the follow-up survey one week later, they reported reaching out to fewer than three old friends during this period ($M = 2.6$, $SD = 2.6$).

Of note, an ANOVA revealed that participants who agreed to be re-contacted for the follow-up survey did not anticipate reaching out to a different number of old friends ($n = 371$; $M = 9.1$, $SD = 15.5$) as compared to those who did not agree to the follow-up survey ($n = 222$; $M = 10.4$, $SD = 18.8$), $F(1, 591) = 0.90$, $p =.34$. Similarly, an ANOVA revealed that participants who responded to the follow-up survey did not anticipate reaching out to a different number of old friends ($n = 191$; $M = 8.6$, $SD = 16.3$) than those participants who agreed to be contacted but ultimately did not respond to the survey ($n = 180$; $M = 9.6$, $SD = 14.7$), $F(1, 369) = 0.38$, $p =.54$.
