# Supplementary material for: People are surprisingly hesitant to reach out to old friends
Source: Commun Psychol. 2024 Apr 23;2:34. doi: 10.1038/s44271-024-00075-8 (PMC11332216; doi:10.1038/s44271-024-00075-8)
Supplement: Supplementary file 3 — Reporting Summary [file 44271_2024_75_MOESM3_ESM.pdf]

nature portfolio

Corresponding author(s): Lara Aknin & Gillian Sandstrom

Last updated by author(s): Feb 16, 2024

Reporting Summary

Nature Portfolio wishes to improve the reproducibility of the work that we publish. This form provides structure for consistency and transparency in reporting. For further information on Nature Portfolio policies, see our Editorial Policies and the Editorial Policy Checklist.

Statistics

For all statistical analyses, confirm that the following items are present in the figure legend, table legend, main text, or Methods section.

n/a	Confirmed	
☐	☒	The exact sample size (*n*) for each experimental group/condition, given as a discrete number and unit of measurement
☐	☒	A statement on whether measurements were taken from distinct samples or whether the same sample was measured repeatedly
☐	☒	The statistical test(s) used AND whether they are one- or two-sided<br>*Only common tests should be described solely by name; describe more complex techniques in the Methods section.*
☒	☐	A description of all covariates tested
☐	☒	A description of any assumptions or corrections, such as tests of normality and adjustment for multiple comparisons
☐	☒	A full description of the statistical parameters including central tendency (e.g. means) or other basic estimates (e.g. regression coefficient) AND variation (e.g. standard deviation) or associated estimates of uncertainty (e.g. confidence intervals)
☐	☒	For null hypothesis testing, the test statistic (e.g. *F*, *t*, *r*) with confidence intervals, effect sizes, degrees of freedom and *P* value noted<br>*Give P values as exact values whenever suitable.*
☐	☒	For Bayesian analysis, information on the choice of priors and Markov chain Monte Carlo settings
☐	☒	For hierarchical and complex designs, identification of the appropriate level for tests and full reporting of outcomes
☐	☒	Estimates of effect sizes (e.g. Cohen's *d*, Pearson's *r*), indicating how they were calculated

Our web collection on statistics for biologists contains articles on many of the points above.

Software and code

Policy information about availability of computer code

Data collection	Data were collected using Qualtrics, an online survey creation and data collection platform.
Data analysis	Data were analyzed using SPSS 28.01 and R version 4.3.2

For manuscripts utilizing custom algorithms or software that are central to the research but not yet described in published literature, software must be made available to editors and reviewers. We strongly encourage code deposition in a community repository (e.g. GitHub). See the Nature Portfolio guidelines for submitting code & software for further information.

Data

Policy information about availability of data

All manuscripts must include a data availability statement. This statement should provide the following information, where applicable:

- Accession codes, unique identifiers, or web links for publicly available datasets
- A description of any restrictions on data availability
- For clinical datasets or third party data, please ensure that the statement adheres to our policy

The manuscript includes the following data availability statement: All materials and data are available on the Open Science Framework (OSF): https://osf.io/kydb3/.

Human research participants

Policy information about studies involving human research participants and Sex and Gender in Research.

Reporting on sex and gender	Participants self-reported their gender. Gender differences were not of particular interest and statistical tests were therefore not conducted.
Population characteristics	There are no covariate-relevant population characteristics.
Recruitment	Participants were recruited in various ways across the nine studies in this manuscript.<br>Study 1: Undergraduate students enrolled for a study to earn course credit. As such, insights may be particularly relevant to younger adults and/or this specific population. However, these limitations are addressed by the broader samples collected in subsequent studies.<br>Study 2: Adults from the United Kingdom and United States were recruited online through a paid participation platform called Prolific.<br>Study 3: Adults from the United Kingdom, United States, and Canada were recruited online through Prolific.<br>Study 4: Adults from the United Kingdom, United States, and Canada were recruited online through Prolific.<br>Study 5: Students, faculty, and staff were recruited in public spaces on a university campus in Canada. This sample includes a broader age range than a student-only sample, but a particularly educated sample.<br>Study 6: Adults from the United Kingdom, United States, and Canada were recruited online through Prolific.<br>Study 7: Students, faculty, and staff were recruited in public spaces on a university campus in the U.K.<br>Study S8: A sample of American adults representative of the United States in age, ethnicity, gender, region, and household income were recruited online through Dynata.<br>Study S9: Students, faculty, and staff were recruited in public spaces on a university campus in Canada. This sample includes a broader age range than student/convenience samples, but a particularly educated sample.
Ethics oversight	Simon Fraser University and University of Sussex

Note that full information on the approval of the study protocol must also be provided in the manuscript.

Field-specific reporting

Please select the one below that is the best fit for your research. If you are not sure, read the appropriate sections before making your selection.

☐ Life sciences ☒ Behavioural & social sciences ☐ Ecological, evolutionary & environmental sciences

For a reference copy of the document with all sections, see nature.com/documents/nr-reporting-summary-flat.pdf

Behavioural & social sciences study design

All studies must disclose on these points even when the disclosure is negative.

Study description	Data from all nine studies are quantitative in nature . The study designs are as follow: survey (Study 1), experiment (Study 2), experiment (Study 3), experiment (Study 4), survey (Study 5), survey (Study 6), experiment (Study 7), survey (Study S8), and survey (Study S9).
Research sample	Study 1: Undergraduate students enrolled for a study to earn course credit. Demographics: Mean age = 19.2, SD = 2.0; 305 women 86 men, 10 other, which is not representative of the student body. Data were a convenience sample.<br>Study 2: Adults from the United Kingdom and United States were recruited online through a paid participation platform called Prolific. Demographics: Mean Age= 27.4, SD = 1.9; 122 women 73 men 4 other, which is not representative of the online participant population. Data were a convenience sample.<br>Study 3: Adults from the United Kingdom, United Stages, and Canada were recruited online through Prolific. Demographics: Mean Age= 39.3, SD = 12.8; 237 women 213 men 3 other, which is not representative of the online participant population. Data were a convenience sample.<br>Study 4: Adults from the United Kingdom, United Stages, and Canada were recruited online through Prolific. Demographics: Mean Age= 40.5, SD = 13.0; 274 women, 327 men 3 other, which is not representative of the online participant population. Data were a convenience sample.<br>Study 5: Students, faculty, and staff were recruited in public spaces on a university campus in Canada. Demographics: Mean age = 20.7, SD = 2.9; 172 women, 107 men, 5 gender fluid/non-binary/both, 4 participants with undisclosed gender, which is not representative of the university population. Data were a convenience sample.<br>Study 6: Adults from the United Kingdom, United Stages, and Canada were recruited online through Prolific. Demographics: Mean Age M = 39.5, SD = 13.4; 138 women, 176 men, 5 other, which is not representative of the online participant population. Data were a convenience sample.<br>Study 7: Students, faculty, and staff were recruited in public spaces on a university campus in the U.K. Demographics: Mean age = 23.2, SD = 7.5; 112 who identified as women, 65 as men, 10 in other ways, and 7 participants with undisclosed gender. Data were a convenience sample.<br>Study S8: A sample of American adults representative recruited on Dynata. Demographics: Mean Age= 43.8, SD = 17.3; 331 identified

	as women, 304 as men, 2 other. The sample was representative of the United States population in terms of age, ethnicity, gender, region, and household income.<br>Study S9: Students, faculty, and staff were recruited in public spaces on a university campus in Canada. Demographics: Mean age = 21.2, SD = 5.0; 120 women, 61 men, 9 other, and 1 participant with undisclosed gender. Data were a convenience sample.
Sampling strategy	Samples were recruited via convenience and paid pools (e.g., Prolific, Dynata). Detailed information regarding sample size determination is contained in the manuscript. However, in short, the sample size for Studies 3-7 and S9 was determined a priori using power calculations to provide at least 80-90% power to detect the smallest effect size of interest. Sample size calculations for Studies 1, 2, and S8 were determined for other empirical questions.
Data collection	All data were collected using the Qualtrics survey platform.
Timing	Our best records indicate studies were run at the following times: Study 1 (January 2022), Study 2 (May 2022), Study 3 (July 2022), Study 4 (December 2022), Study 5 (February-March 2023), Study 6 (November 2023), Study 7 (October-November 2023), Study S8 (December 2022), Study S9 (October-November 2023).
Data exclusions	The number and rationale for all exclusions is reported in the manuscript.
Non-participation	The number of people who chose not to participate is reported where appropriate and available.
Randomization	In Studies 2, 3, 4, and 7 (all those involving random assignment to condition), random assignment was determined by the Qualtrics survey programming.

Reporting for specific materials, systems and methods

We require information from authors about some types of materials, experimental systems and methods used in many studies. Here, indicate whether each material, system or method listed is relevant to your study. If you are not sure if a list item applies to your research, read the appropriate section before selecting a response.

Materials & experimental systems

n/a	Involved in the study	
☒	☐	Antibodies
☒	☐	Eukaryotic cell lines
☒	☐	Palaeontology and archaeology
☒	☐	Animals and other organisms
☒	☐	Clinical data
☒	☐	Dual use research of concern

Methods

n/a	Involved in the study	
☒	☐	ChIP-seq
☒	☐	Flow cytometry
☒	☐	MRI-based neuroimaging
